# *In vitro *model for the analysis of synovial fibroblast-mediated degradation of intact cartilage

**DOI:** 10.1186/ar2618

**Published:** 2009-02-18

**Authors:** David Pretzel, Dirk Pohlers, Sönke Weinert, Raimund W Kinne

**Affiliations:** 1 Experimental Rheumatology Unit, Department of Orthopedics, University Hospital Jena, Friedrich Schiller University Jena, Klosterlausnitzer Strasse 81, Eisenberg, D-07607, Germany; 2 Current address: Experimental Cardiology, Otto von Guericke University Magdeburg, Leipziger Strasse 44, Magdeburg, D-39120, Germany

## Abstract

**Introduction:**

Activated synovial fibroblasts are thought to play a major role in the destruction of cartilage in chronic, inflammatory rheumatoid arthritis (RA). However, profound insight into the pathogenic mechanisms and the impact of synovial fibroblasts in the initial early stages of cartilage destruction is limited. Hence, the present study sought to establish a standardised *in vitro *model for early cartilage destruction with native, intact cartilage in order to analyse the matrix-degrading capacity of synovial fibroblasts and their influence on cartilage metabolism.

**Methods:**

A standardised model was established by co-culturing bovine cartilage discs with early-passage human synovial fibroblasts for 14 days under continuous stimulation with TNF-α, IL-1β or a combination of TNF-α/IL-1β. To assess cartilage destruction, the co-cultures were analysed by histology, immunohistochemistry, electron microscopy and laser scanning microscopy. In addition, content and/or neosynthesis of the matrix molecules cartilage oligomeric matrix protein (COMP) and collagen II was quantified. Finally, gene and protein expression of matrix-degrading enzymes and pro-inflammatory cytokines were profiled in both synovial fibroblasts and cartilage.

**Results:**

Histological and immunohistological analyses revealed that non-stimulated synovial fibroblasts are capable of demasking/degrading cartilage matrix components (proteoglycans, COMP, collagen) and stimulated synovial fibroblasts clearly augment chondrocyte-mediated, cytokine-induced cartilage destruction. Cytokine stimulation led to an upregulation of tissue-degrading enzymes (aggrecanases I/II, matrix-metalloproteinase (MMP) 1, MMP-3) and pro-inflammatory cytokines (IL-6 and IL-8) in both cartilage and synovial fibroblasts. In general, the activity of tissue-degrading enzymes was consistently higher in co-cultures with synovial fibroblasts than in cartilage monocultures. In addition, stimulated synovial fibroblasts suppressed the synthesis of collagen type II mRNA in cartilage.

**Conclusions:**

The results demonstrate for the first time the capacity of synovial fibroblasts to degrade intact cartilage matrix by disturbing the homeostasis of cartilage via the production of catabolic enzymes/pro-inflammatory cytokines and suppression of anabolic matrix synthesis (i.e., collagen type II). This new *in vitro *model may closely reflect the complex process of early stage *in vivo *destruction in RA and help to elucidate the role of synovial fibroblasts and other synovial cells in this process, and the molecular mechanisms involved in cartilage degradation.

## Introduction

Rheumatoid arthritis (RA) is a chronic disorder primarily affecting the joints and leading to destruction of the articular cartilage with subsequent severe morbidity and disability. It is characterised by a chronic infiltration of inflammatory cells into the synovial membrane and the development of a hyperplastic pannus tissue [1].

This pannus tissue, consisting of both inflammatory and resident mesenchymal cells, invades and destroys the underlying cartilage and bone. Therefore, the role of macrophages [2], T- and B-cells [3] and synovial fibroblasts (SFB) [4] in the pathogenesis of RA, including their multilateral interactions, has been intensely investigated. Due to their aggressive features and over-expression of matrix-degrading enzymes, activated SFB seem to play a major role in the invasion and proteolytic degradation of the cartilage matrix [5]. In addition, they can indirectly induce a catabolic metabolism in chondrocytes via soluble mediators [6]. The destructive properties of SFB have been analysed in several *in vivo *and *in vitro *models. Despite their unquestionable advantages, established animal models of arthritis, including co-implantation models in immunodeficient mice (reviewed in [7,8]), also have disadvantages. They reflect a very complex cellular network rather than the particular influence of a certain cell type, are time-consuming and expensive, and can be ethically problematic.

In an attempt to replace, or at least reduce, the number of animal experiments, several co-culture models of cartilage destruction have been established to date. Besides differences in the co-cultured cell types and their purity (whole synovial membranes, pools of synovial macrophages, fibroblasts, T- and B-cells, or polymorphic neutrophilic leucocytes), most notably the type of cartilage (-like) matrix varied widely. The types of cartilage ranged from artificial, cell-free matrix substitutes based on collagen/peptide matrices [9] or extracted cartilage components (reconstituted from milled cartilage) [10] to *in vitro *generated, cell-containing matrices (derived from the three-dimensional (3D) culture of chondrocytes) [11]. In artificial matrices, however, the matrix structure barely resembles the natural structure and properties of native cartilage concerning zonal architecture, density, rigidity and composition of matrix constituents. In the case of *in vitro *models with isolated chondrocytes, on the other hand, cells may de-differentiate from their chondrogenic phenotype (even in 3D culture) and a re-differentiation of the expanded chondrocytes may be difficult to achieve, especially in long-term cultures.

Therefore, some research groups have used native cartilage explants (mostly human) for studies on the matrix-degrading capacities of synovial cells [12,13]. However, the human cartilage available via joint replacement surgery is from patients with severe osteoarthritis (OA) or RA and is mostly of poor quality and shows a high heterogeneity of the pre-existing cartilage erosions, so standardisation for *in vitro *models is difficult.

The objective of the present study, therefore, was to establish a standardised *in vitro *model of RA-related early cartilage destruction with native, intact cartilage in order to analyse the matrix-degrading capacity of SFB and their influence on the cartilage metabolism. Purified, early-passage SFB were used in co-culture with cartilage to reduce the complex cellular network to the main elements of interest. The focus of the model was the representation of initial cartilage destruction, thereby reflecting the main features of early matrix degradation in RA under well-defined and reproducible conditions.

For this purpose, a 48-well plate *in vitro *system was established, consisting of an interactive co-culture of bovine cartilage discs with isolated RA SFB. In addition, the system was stimulated with TNF-α and IL-1β (two pro-inflammatory cytokines centrally involved in the pathogenic process of RA) in order to simulate the influence of macrophage (leukocyte)-derived pro-inflammatory cytokines on both chondrocytes and SFB *in vivo*.

Cartilage destruction was monitored by histological and immunohistological methods and tissue-degrading enzymes, as well as pro-inflammatory cytokines in both SFB and chondrocytes were studied on a transcriptional and protein level.

## Materials and methods

### Isolation and culture of synovial fibroblasts

Synovial tissue was obtained during synovectomy from patients with RA in the Orthopedic Clinic, Waldkrankenhaus 'Rudolf Elle' Eisenberg, Germany. All patients fulfilled the American Rheumatism Association criteria for RA [14] and had given their informed consent (for additional patient information see Table 1). The study was approved by the Ethics Committee of the Friedrich Schiller University. Negative purification of SFB from primary culture synovial cells was carried out as previously described (purity ≥ 98%) [15].

**Table 1:** Clinical characteristics of the patients at the time of synovectomy

Patients (total)	Gender (male/female)	Age (years)	Disease duration (years)	RF (titre)	ESR (mm/hour)	CRP (mg/l)	Number of ARA criteria	Concomitant medication
5	3/2	6.18 ± 4.4	9.6 ± 2.6	100 ± 33.7	49.4 ± 11.8	47.6 ± 12.3	5.6 ± 0.5	MTX (2) NSAID (4)

For the parameters age, disease duration, erythrocyte sedimentation rate (ESR), C-reactive protein (CRP; normal range, < 5 mg/l) and number of American Rheumatism Association (ARA) criteria, means ± standard error of the mean are given. MTX = methotrexate; NSAID = non-steroidal anti-inflammatory drugs; RF = rheumatoid factor.

Frozen and subsequently thawed SFB (first passage) were cultured to 80 to 100% confluency in SFB-medium (Dulbecco's modified eagle media (DMEM) containing 100 μg/ml gentamycin, 100 μg/ml penicillin/streptomycin, 20 mM 4-(2-hydroxyethyl)-1-piperazineethanesulfonic acid and 10% FCS). As a preparation for the co-culture experiments, SFB were cultured for 24 hours before co-culture with a medium mixture containing equal parts of SFB medium and co-culture medium (DMEM and F12 Nutmix; ratio 1:1 (Invitrogen, Karlsruhe, Germany), containing 100 μg/ml gentamycin, 5% FCS, and ITS-culture supplement (1:1000; final concentrations: 5 μg/ml insulin and transferrin, 5 ng/ml selenic acid; BD Biosciences, Heidelberg, Germany)).

### Preparation and embedding of bovine cartilage

**Figure 1:**
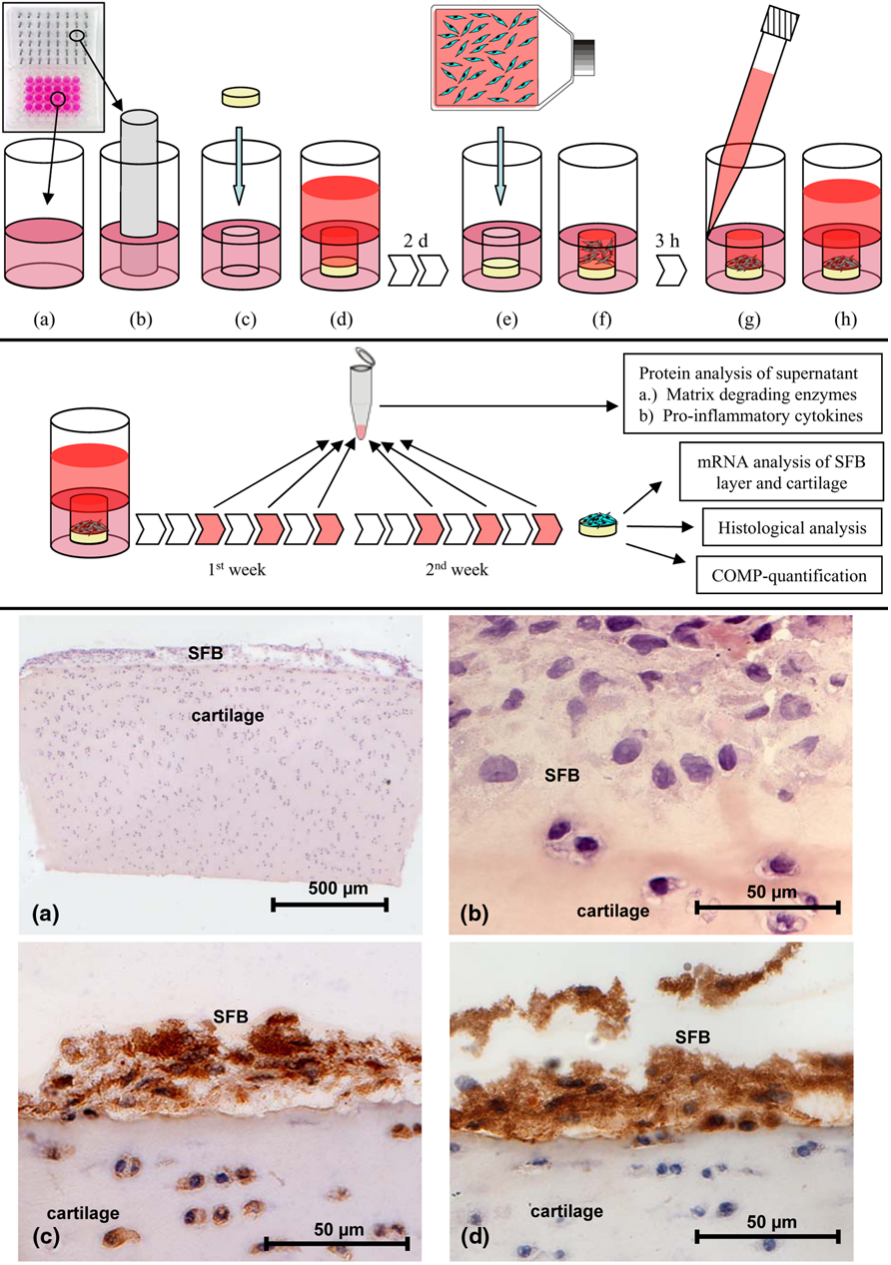
Experimental setup of the in vitro model and histological characterization of the cartilage co-cultured with SFB. Upper panel: Embedding of cartilage and subsequent co-culture with synovial fibroblasts (SFB). **(a)** Hot 2% agarose was filled in each well of a 48-well plate and **(b)** a cylinder was created in the agarose by inserting a metal pin plate and removing the plate after polymerisation. **(c-d) **Subsequently cartilage disc were embedded in the preformed cylinder and pre-cultured for two days. **(e) **The SFB suspension was then applied and **(f) **left for three hours for settling and initial adherence of the SFB on the cartilage surface. Finally, **(g-h)** co-culture medium was carefully added into the upper well compartment. Middle panel: Experimental setup. Cultures were maintained for two weeks, medium was replaced every two to three days, and supernatants were collected and subjected to protein analysis. Cultured constructs were either further processed for histological evaluation and quantification of cartilage oligomeric matrix protein (COMP) content in cartilage or used for gene expression analysis of SFB and cartilage. Lower panel: Histological and immunohistochemical staining of cartilage co-cultures with SFB after 14 days of *in vitro *culture. **(a, b)** H&E staining demonstrates the formation of a SFB multilayer on the cartilage surface. **(c)** Immunostaining for prolyl-4-hydroxylase verifies the viability of SFB and chondrocytes and **(d)** immunostaining for human Thy-1 proves the fibroblast origin of the co-cultivated cells. Magnification (a) 40×, (b) 630× and (c, d) 400×.

Cartilage was obtained on the day of slaughter from bovine knee joints (German Holstein Friesian Cattle; average age 24 months). Cartilage discs were aseptically dissected from the lateral sites of the facies articularis of the bovine femur using a biopsy punch (inner diameter 3 mm) and a scalpel (resulting height of the discs 1.3 ± 0.3 mm). The cartilage discs were directly transferred into a dish containing co-culture medium. The cartilage discs obtained from different locations were randomly distributed to the different experimental groups. For embedding of the discs, a total of 450 μl hot, liquid, 2% agarose (normal melting point; Invitrogen) was filled into the wells of a 48-well plate. Cylinders of a defined size were created by inserting a self-manufactured metal-pin plate into the hot agarose (Figures 1a, b; upper panel). The cartilage discs were then embedded in the preformed cylinders with the intact surface orientated upside (Figure 1c; upper panel). Afterwards, the wells were filled with 300 μl co-culture medium and kept in an atmosphere of 37°C, 5% carbon dioxide for 48 hours (Figure 1d; upper panel).

This was performed to ensure the reliable fixation of the cartilage discs on the bottom of the pre-formed cylinders, to create a defined space above the discs for subsequent application and seeding of the SFB exclusively on the cartilage surface and to reduce the shear forces acting on the co-culture system during media exchange (Figures 1e to 1h; upper panel). The use of agarose, on the other hand, allowed sufficient diffusion of nutrients from the medium into the embedded cartilage matrix.

### Cartilage co-culture with synovial fibroblasts

Co-culture medium was removed from the cartilage pre-culture and 25 μl of the trypsin-treated SFB suspensions (n = 5 separate RA-SFB cultures; 2 × 10^4 ^cells each) in the 1:1 medium mixture were carefully added drop-wise onto the cartilage surface. After three hours of co-culture, 550 μl co-culture medium with/without TNF-α (10 ng/ml), IL-1β (5 ng/ml) or a combination of TNF-α/IL-1β (PeproTech, Hamburg, Germany) were added to the well. These cytokine concentrations represent the dose of each cytokine with the maximum effect in monocultures of stimulated SFB (concerning the induction of several matrix-metalloproteinase (MMP), as determined in initial experiments, data not shown). The co-culture was then continued for 14 days at 37°C and 5% (v/v) carbon dioxide. Every two to three days, 550 μl of the culture supernatants were carefully removed for analysis and replaced with fresh co-culture medium with/without cytokines. Supernatants were pooled over two weeks and stored at -20°C for further analyses (Figure 1; central panel).

In each experimental group, six replicates were cultured in parallel, four were analysed histologically and two were processed for mRNA analysis of the SFB layer and cartilage. For each experimental parameter, patient SFB were analysed separately for each donor.

After 14 days of *in vitro *co-culture, multiple layers of SFB were observed exclusively on the intact cartilage surface (but not on the adjacent cutting edges; Figure 1a, lower panel). SFB and chondrocytes remained vital (except for the chondrocytes close to the lateral edges, probably as a result of the compression by the biopsy punch), as shown by positive staining with prolyl-4-hydroxylase (Figure 1b, lower panel) and mRNA production for several molecules. To ensure that the cell layers on top of the cartilage surface were formed by SFB and not by migrated chondrocytes, immunohistochemistry for the human-specific fibroblast marker Thy-1 (CD90) was used. According to this marker, human SFB formed a distinct layer on the cartilage, whereas chondrocytes in the cartilage matrix were not stained at all (Figure 1c, lower panel).

### Labelling of synovial fibroblasts for analysis by laser scanning microscopy

Twenty-four hours before co-culture, SFB were labelled with 5 μM 5-(and-6)-carboxyfluorescein diacetate, succinimidyl ester (CFSE; Molecular Probes, Karlsruhe, Germany) according to the supplier's instructions. This fluorescent dye becomes impermeable to cell membranes after cellular intake and remains trapped intracellularly for the whole co-culture period of two weeks. Invasion of SFB into cartilage matrix was analysed in a wet state after two weeks of co-culture using a laser scanning microscope LSM 510 Meta (Carl Zeiss, Jena, Germany). Filters were chosen according to the emission wavelength of the CFSE dye (λ_ex _= 488 nm and λ_em _= 530 nm). In addition, the reflection signal of the unlabelled cartilage was measured in a second detector channel.

### Histology and immunohistochemistry

Fresh, non-cultured cartilage discs or cultured cartilage discs with/without SFB were embedded in tissue freezing medium (Leica, Nussloch, Germany) and immediately frozen in 2-methyl-butane cooled with liquid nitrogen. Cryosections (8 μm) were mounted on aminoalkyl-silane-coated slides. Cartilage and SFB morphology was analysed after conventional H&E staining (Hollborn, Leipzig, Germany). Proteoglycan loss from cartilage was quantified after staining with safranin-O and counterstaining with light green at low magnification (40×) using the image analysing software DatInfMeasure (DatInf GmbH, Tübingen, Germany) and by measuring the total area and the safranin-O positive/negative areas.

For immunohistochemistry, frozen sections were dried overnight and fixed for 10 minutes either in acetone (anti-prolyl-4-hydroxylase and anti-Thy-1 monoclonal antibodies) or in 4% paraformaldhyde in PBS. Endogenous peroxidase activity was blocked with 0.5% hydrogen peroxide in ethanol. Demasking of epitopes (cartilage oligomeric matrix protein (COMP) and COL2-3/4-C (short)) was performed by incubation with chondroitinase ABC (Sigma-Aldrich, Deisenhofen, Germany). After blocking nonspecific binding sites with 10% rabbit or goat serum (same species as the source of the secondary antibody) in PBS, sections were incubated for one hour with primary antibodies against prolyl-4-hydroxylase (Biomeda, Foster City, CA, USA), Thy-1 (CD90; Dianova, Hamburg, Germany), COMP (rabbit polyclonal antibody directed against human and bovine COMP; Kamiya Biomedicals, Seattle, WA, USA) or the collagen cleavage epitope Col2-3/4C-C (short) (immunoreactive with human and bovine epitopes, TECO Medical, Sissach, Switzerland) and, subsequently, with horseradish peroxidase (HRP)-conjugated rabbit anti-mouse immunoglobulin (Ig) G/or goat anti-rabbit IgG (Santa Cruz Biotechnology, Santa Cruz, CA, USA). The peroxidase was revealed using diaminobenzidine or 3-amino-9-ethylcarbazole (both Sigma-Aldrich). Slides were counterstained with haematoxylin and covered with Aquatex (Merck, Darmstadt, Germany). Mouse IgG_1_/IgG_2a _(DAK-GO1/DAK-GO5; both from Dako, Glostrup, Denmark) or affinity-purified rabbit IgG (Sigma-Aldrich) served as isotype controls and always yielded negative results.

### Transmission electron microscopy

Cartilage discs were chemically pre-fixed for 24 hours at room temperature (4% glutaraldehyde; 0.1 M sodium cacodylate buffer; pH 7.2; Roth, Karlsruhe, Germany), post-fixed for 24 hours (1% osmium tetroxide; 0.1 M sodium cacodylate buffer, pH 7.4), rinsed three times in isotonic buffer solution (0.1 M sodium cacodylate buffer, pH 7.2), and finally transferred to 100% acetone by dehydration through a graded series of acetone. Discs were then incubated with 2% uranyl acetate for one hour, washed with propylene oxide and embedded in araldite by polymerisation at 60°C. Vertical, semi-thin sections were cut on a Leica Ultracut E ultramicrotome and stained for 15 minutes in 1% Richardson solution (Hollborn). Subsequently, thin sections were cut (about 60 nm thick), mounted on copper grids, stained for five minutes with a mixture of 80 mM sodium citrate, 40 mM lead nitrate and 40 mM sodium hydroxide, and examined on a Philips CM 10 transmission electron microscope (Philips, Hamburg, Germany). Transmission electron microscopy of cartilage is known to illustrate the collagen network structure, whereas proteoglycans are collapsed and not visible due to the fixation method.

### RNA isolation

**Figure 2:**
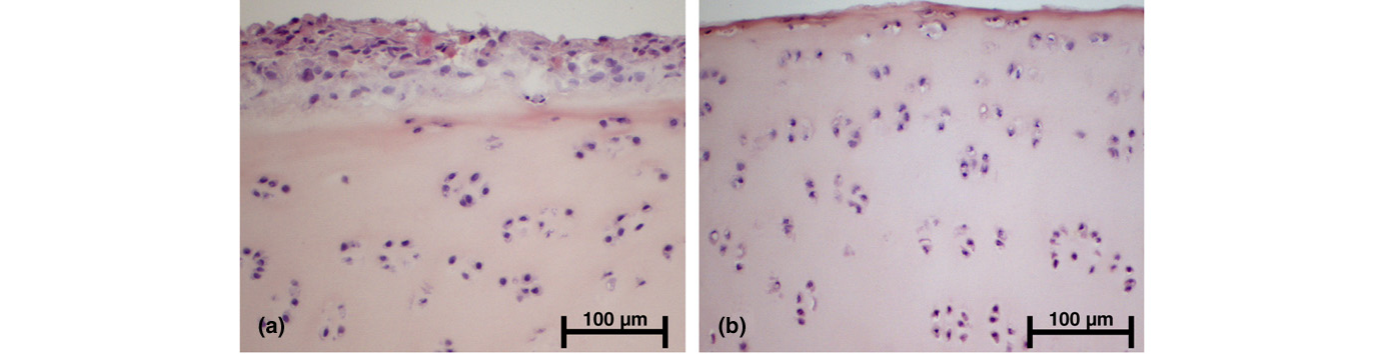
Histological analysis of cartilage co-cultures with synovial fibroblasts (SFB) **(a)** before and **(b) **after detachment of SFB by short incubation with lysis buffer. SFB were completely removed from the cartilage surface, whereas cartilage matrix and chondrocytes remained intact. H&E staining, magnification 200×.

The SFB layer was carefully detached from the cartilage disc by incubating the cartilage/SFB composite for 10 seconds in 75 μl RLT-lysis buffer (RNeasy^® ^Micro kit; Qiagen, Hilden, Germany) containing 15 ng carrier RNA. This procedure completely removed the SFB from the cartilage surface, but left the chondrocytes in the cartilage intact as assessed by histological analysis (Figure 2) and quantitative PCR (qPCR) using species-specific primers (data not shown). Total RNA was then isolated using the RNeasy^® ^Micro kit according to the supplier's instructions including a DNase digestion step.

Following removal of the SFB (in the case of co-culture), the shock-frozen cartilage was pulverised in a microdismembrator (Braun, Melsungen, Germany) by milling it for 30 seconds with an agitated grinding ball in a liquid nitrogen-cooled, stainless steel vessel (shaking rate of 2000 per minute and amplitude of 16 mm). Subsequently, RNA was extracted by resuspension of the powder in 400 μl RLT-lysis buffer containing carrier RNA and centrifugation. After addition of 800 μl RNase-free water, interfering matrix components were removed by digesting the supernatant for 10 minutes at 55°C with proteinase K (20 mg/ml; Qiagen). Total RNA was then isolated as above. This method enabled us to isolate intact RNA from small cartilage samples (about 5 mg per preparation) with a good yield. The integrity of the RNA samples was demonstrated by detection of distinct 28S and 18S rRNA bands without smear by agarose gelelectrophoresis in selected samples (data not shown).

### Reverse transcription and quantitative PCR

Total RNA eluate (10 μl) was primed with oligo(d)T and reverse-transcribed for one hour at 42°C using SuperScript-II reverse transcriptase (Invitrogen).

qPCR reactions were performed as previously described [16] with cloned standards for the quantitation of human MMP-1, MMP-3, IL-6, IL-8, and the housekeeping gene aldolase and a batch preparation of bovine cDNA for the cartilage samples. qPCR was performed on a mastercycler 'realplex2' (Eppendorf, Hamburg, Germany) with HotMaster Taq (Eppendorf) and the primer pairs and PCR conditions presented in Table 2. The relative concentrations of cDNA present in each sample were calculated by the software using the standard curves. In order to normalise the amount of cDNA in each sample and to guarantee the comparability of the calculated mRNA expression in all analysed samples, the housekeeping gene aldolase was amplified. Product specificity was confirmed by melting curve analysis and initial cycle sequencing of the PCR products.

**Table 2:** Primers, product length and specific amplification conditions for qPCR

**Gene**	**Primer forward**	**Primer reverse**	**Accession number**	**T annealing**	**Melting T product**
**Human/bovine Aldolase A**	5'-TCATCCTCTTCCATGAGACACTCTA-3'	5'ATTCTGCTGGCAGATACTGGCATAA-3'	[GenBank: NM_000034]	58°C	88°C
**Human MMP-1**	5'-GACCTGGAGGAAATCTTGC-3'	5'-GTTAGCTTACTGTCACACGC-3'	[GenBank: NM_002421]	58°C	86°C
**Human MMP-3**	5'-CTCACAGACCTGACTCGGTT-3'	5'-CACGCCTGAAGGAAGAGATG-3'	[GenBank: NM_002422]	58°C	81°C
**Human IL-6**	5'-ATGAACTCCTTCTCCACAAGCG-3'	5'-CTCCTTTCTCAGGGCTGAG-3'	[GenBank: NM_000600]	60°C	86°C
**Human IL-8**	5'-GCCAAGAGAATATCCGAACT-3'	5'-AGGCACAGTGGAACAAGGACTTGT-3'	[GenBank: NM_000584]	60°C	78°C
**Bovine MMP-1**	5'-CAAGAGCAGATGTGGACCAA-3'	5'-CTGGTTGAAAAGCATGAGCA-3'	[GenBank: NM_174112]	61°C	83°C
**Bovine MMP-3**	5'-CTGGTGTCCAGAAGGTGGAT-3'	5'-TAGGCGCCCTTGAAGAAGTA-3'	[GenBank: AB043995]	61°C	83°C
**Bovine IL-6**	5'-ATGAACTCCCGCTTCACAAG-3'	5'-CCTTGCTGCTTTCACACTCA-3'	[GenBank: NM_173923]	61°C	83°C
**Bovine IL-8**	5'-TGCTCTCTGCAGCTCTGTGT-3'	5'-CAGACCTCGTTTCCATTGGT-3'	[GenBank: NM_173925]	64°C	81°C
**Bovine Col II (α 1 chain)**	5'-CATCTGGTTTGGAGAAACCATC-3'	5'-GCCCAGTTCAGGTCTCTTAG-3'	[GenBank: NM_001001135]	61°C	83°C
**Bovine COMP**	5'-ATGCGGACAAGGTGGTAGAC-3'	5'-TCTCCATACCCTGGTTGAGC-3'	[GenBank: X74326]	61°C	87°C

General amplification protocol (40 cycles): initial denaturation for two minutes at 95°C; denaturation for 15 seconds at 95°C, specific primer annealing temperature (see table) for 15 seconds, amplification at 68°C for 20 seconds, additional heating step to 5°C below the melting temperature of the PCR product (see table). General melting curve protocol (one cycle): denaturation for one second at 95°C; cooling to 5°C above the primer annealing temperature (holding for 10 seconds); heating to 95°C (0.1°C/second); final cooling for five minutes at 40°C.

Col = collagen; COMP = cartilage oligomeric matrix protein; IL = interleukin; MMP = matrix metalloproteinase; qPCR = quantitative polymerase chain reaction.

### MMP-activity assay

The synthetic peptide Mca-Pro-Leu-Gly-Leu-Dap(Dnp)-Ala-Arg-NH_2 _(Bachem, Heidelberg, Germany) was used to quantify the sum activity of bovine and human MMP in pooled supernatants (two weeks of culture). This fluorogenic substrate peptide is a very sensitive substrate for the *in situ *determination of the MMP activity. Cleavage at the Gly-Leu bond separates the highly fluorescent Mca group from the efficient 2,4-dinitrophenyl quencher, resulting in an increase of fluorescence intensity. The substrate peptide can be cleaved by numerous MMP, with MMP-2, MMP-9 and, to a lesser extent, MMP-1, MMP-3 and MMP-13 showing the highest rates of turnover [17]. To estimate the potential total activity of latent and active MMP, latent MMP were activated by incubation with 2 mM aminophenylmercuric acetate (APMA; Sigma-Aldrich); without APMA activation, none of the samples showed any MMP activity.

For the assay, 10 μl culture-supernatant were incubated for two hours at 37°C with 20 μl of 25 μM MCA-Pro-Leu-Gly-Leu-DAP(DNP)-Ala-Arg-NH_2 _in 70 μl incubation buffer (100 mM Tris/HCl, 30 mM calcium chloride, 1 μM zinc chloride_, _2 mM APMA, 0.05% Brij, pH 7.6) and the increase of the fluorescence intensity was measured at 390 nm. Fresh, co-culture medium containing FCS was analysed as an internal control for MMP activity. Although the values in the medium control were only marginally higher than those in the buffer control, the values in the co-culture medium were nevertheless subtracted from the values in the experimental samples in order to correct for background MMP activity.

### Casein zymography

Caseinolytic activity in pooled supernatants was assayed by electrophoresis in polyacrylamide gels containing sodium dodecyl sulfate (SDS) and casein (Sigma-Aldrich) using a batch of HT1080-conditioned media as a standard [18]. Fresh co-culture medium served as an internal control for the caseinolytic activity derived from the supplemented FCS. The MMP suggested on the basis of their known caseinolytic activity and the molecular weight of their latent and active forms were then identified by western blot analysis of the same pooled supernatants.

### Western Blot for bovine/human MMP-1 and MMP-3

Pooled culture supernatants (20 μl) were resolved by native SDS-PAGE. MMP-1 was detected by immunoblotting using a primary antibody against active/latent MMP-1 (MAB901, R&DSystems, Wiesbaden, Germany) and goat-anti-mouse IgG HRP as a secondary antibody (Sigma-Aldrich). The blots were stripped and re-probed with primary antibody against active/latent MMP-3 (MAB 513, R&DSystems).

### Enzyme-linked immunosorbent assay

In the supernatants of cartilage cultures with SFB, levels of SFB-derived active/latent MMP-1 were measured using a mouse-anti-human MMP-1 monoclonal antibody (MAB901, R&DSystems) as a capture antibody (1 μg/ml), biotinylated goat-anti-human MMP-1 (BAF901, R&DSystems) as a detector antibody (200 ng/ml) and recombinant human MMP-1 (901-MP-010, R&D Systems) as a standard (39 to 5000 pg/ml). SFB-derived active/latent MMP-3 levels were determined using the anti-human MMP-3 Total Duo Set (R&D Systems), and the levels of SFB-derived IL-6 and IL-8 were analysed using anti-human OptEIA-ELISA Sets (BD Biosciences). Combined aggrecanase I/II activity (reflecting both SFB-derived human and cartilage-derived bovine activity) in the supernatants of cartilage cultures with/without SFB was measured according to the manufacturer's instructions using a commercially available ELISA-Kit (Invitek, Berlin, Germany).

For all enzyme-linked immunosorbent assay (ELISA) measurements, fresh co-culture medium was also analysed for the content of the corresponding molecule in the supplemented serum. Although the values in the medium control were only marginally higher than those in the buffer control, the values in the co-culture medium were nevertheless subtracted from the values in the experimental samples.

### Extraction and quantification of COMP from bovine cartilage

COMP was isolated from cartilage according to the method of DiCesare *et al. *[19] with minor modifications. Briefly, 20 mg of shock-frozen cartilage from monoculture/co-culture with SFB was pulverised according to the procedure described above for RNA isolation; in the case of samples from co-culture experiments, a step with lysis of the SFB layer and subsequent PBS washing of the remaining cartilage was included. The pulver was transferred to a tube with 500 μl ice-cold neutral salt buffer (10 mM Tris/hydrochloric acid, 0.15 M sodium chloride, pH 7.4, containing 1 mM phenylmethylsulfonyl fluoride, 0.025 mg/ml leupeptin, 0.025 mg/ml aprotinin and 0.025 mg/ml pepstatin). After centrifugation, the supernatant was decanted and the tissue was re-suspended in the same buffer. The extraction procedure was completed by two cycles of centrifugation and addition of neutral salt buffer containing 10 mM EDTA. Aliquots (10 μl) of all extracts were analysed by non-reducing and reducing SDS/PAGE. Western blots were developed using a polyclonal rabbit antibody against COMP (same antibody as used for immunohistochemistry) and an HRP-conjugated anti-(rabbit IgG) as a secondary antibody. The major proportion of COMP (pentameric, oligomeric and monomeric, as well as degraded COMP) was enriched in the first neutral salt buffer extract, although only small amounts were detected in the second neutral salt buffer extract and the subsequent two extracts with EDTA-containing buffer (data not shown). The content of cartilage-derived COMP was analysed in pooled extracts using a bovine-specific ELISA-Kit (Anamar Medical, Gothenburg Sweden) according to manufacturer's instructions. The polyclonal antibody used in this assay detected the same COMP species as the polyclonal antibody employed for western blots (personal communication; Anders Sjödin, Anamar Medical) and, therefore, the results represent the overall COMP content in the cartilage matrix expressed as units/mg cartilage.

### Statistics

Analyses were performed using the Mann-Whitney U test and the statistical software SPSS/Win version 10.0 (SPSS, Chicago, USA); differences with p ≤ 0.05 were considered to be statistically significant.

## Results

### Proteoglycan release from cartilage

**Figure 3:**
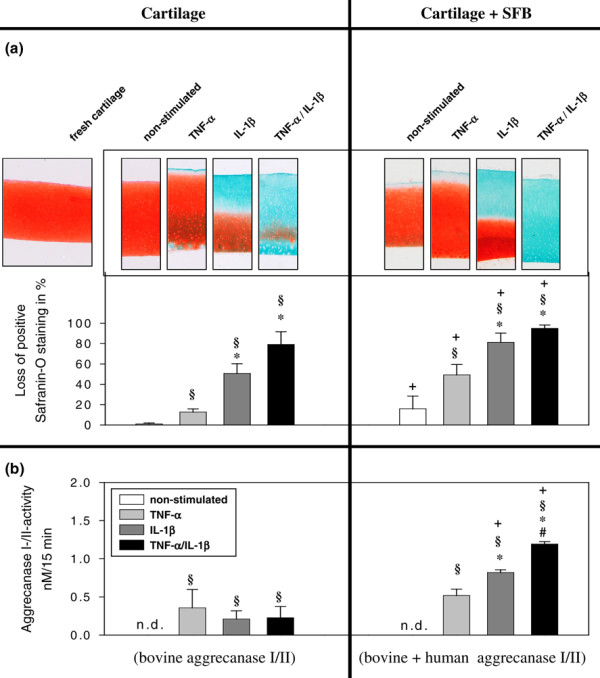
Analysis of proteoglycan loss from cartilage monocultures and co-cultures with SFB. Cartilage monocultures (n = 5, with four replicates each) and co-cultures with SFB (n = 5 patients with four replicates for each patient) with or without stimulation with TNF-α, IL-1β or TNF-α/IL-1β (14 days), as detected by safranin-O staining. **(a) **The upper panel shows representative histological samples, in which red colour indicates the presence and green colour the absence of proteoglycans in the cartilage matrix. Fresh, non-cultured cartilage serves as a positive control. The lower chart depicts the results of quantitative image analysis of the stained sections. **(b)** Aggrecanase I/II activity in culture supernatants of cartilage monocultures and co-cultures with SFB (n = 5 with four replicates for each patient). Mean ± standard error of the mean (SEM) are plotted. § p ≤ 0.05 Mann-Whitney U Test compared with non-stimulated control; * p ≤ 0.05 Mann-Whitney U Test compared with stimulation with TNF-α; # p ≤ 0.05 Mann-Whitney U Test compared with stimulation with IL-1β; + p ≤ 0.05 Mann-Whitney U Test compared with cartilage-monoculture.

Strong safranin O staining was observed in sections of freshly isolated cartilage or in non-stimulated cartilage monocultures, demonstrating minimal loss of proteoglycan after two weeks of *in vitro *culture (about 1%; Figure 3a, left panel).

TNF-α stimulated cartilage was characterised by a slight, but significant proteoglycan loss (10%; Figure 3a, left panel) exclusively at the cartilage surface. This was significantly enhanced in IL-1β stimulated samples, in which a drastic proteoglycan release (50%) occurred in the upper half of the cartilage matrix. In TNF-α/IL-1β stimulated cartilage the proteoglycan loss was higher than the sum of the individual effects (80%; p ≤ 0.05 versus TNF-α), indicating a synergistic effect of the two cytokines.

In comparison to cartilage monocultures, strikingly, non-stimulated cartilage co-cultures with RA-SFB showed a significantly stronger depletion of proteoglycan from the cartilage matrix (15% versus 1%; Figure 3a, right panel). As in the case of monocultures, also the proteoglycan depletion in co-cultures was augmented by stimulation with TNF-α and further enhanced by IL-1β or the combination of TNF-α/IL-1β (both p ≤ 0.05 versus TNF-α; Figure 3a, right panel).

A considerable contribution of the SFB, whether direct or indirect, was demonstrated by the fact that all co-cultures showed a significantly higher proteoglycan depletion than the respective monocultures (Figure 3a, compare left and right panel).

### Aggrecanase activity in the supernatant

There was no aggrecanase activity in non-stimulated cartilage monocultures. Stimulation with TNF-α, IL-1β or the combination of TNF-α/IL-1β led to a similar, significant induction of aggrecanase activity (0.21 to 0.36 nM/15 minutes; Figure 3b, left panel).

As in the case of monocultures, there was no aggrecanase activity in non-stimulated cartilage co-cultures. Again, stimulation with TNF-α and, in particular, IL-1β led to a significant induction of aggrecanase activity (0.52 and 0.82 nM/15 minutes, respectively; Figure 3b, right panel). The aggrecanase activity in the supernatants of double-stimulated co-cultures was significantly higher compared with that after stimulation with either TNF-α or IL-1β.

Interestingly, the aggrecanase activity in cartilage co-culture with SFB was either numerically (for TNF-α) or significantly higher (for IL-1β and TNF-α/IL-1β) than in the corresponding monoculture (Figure 3b, compare left and right panel), again pointing to a contribution of SFB.

### COMP detection in cartilage

**Figure 4:**
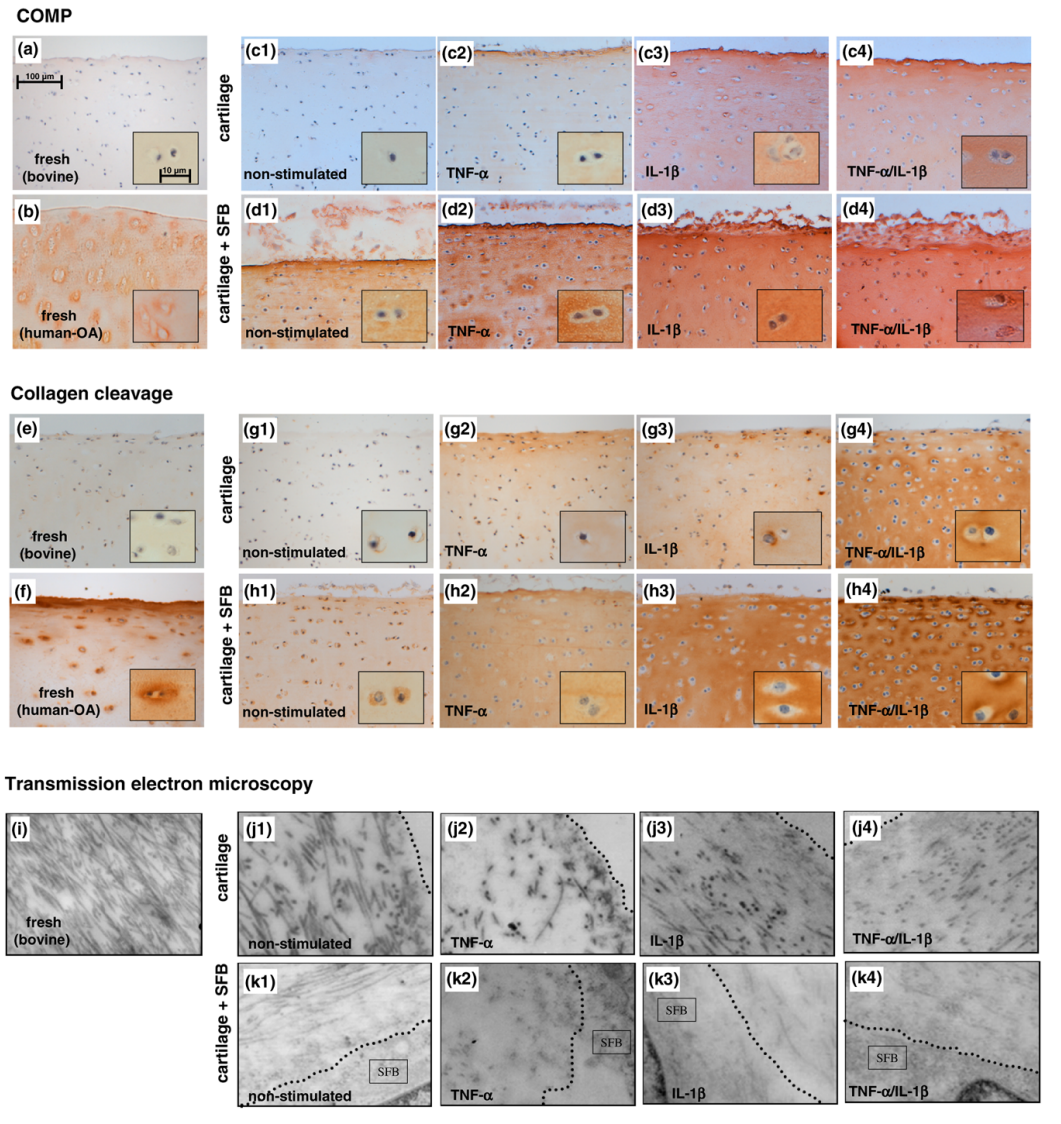
Immunohistochemical staining and electron microscopy. **(a, e, i)** Fresh, non-cultured bovine cartilage and **(b, f)** human osteoarthritis (OA) cartilage, as well as **(c1** to **c4**, **g1** to **g4**, **j1** to **j4)** bovine cartilage from monocultures or **(d1** to **d4**, **h1** to **h4**, **k1** to **k4)** co-cultures with synovial fibroblast (SFB) after 14 days are shown. Immunostaining for cartilage oligomeric matrix protein (COMP) clearly reveals a (c1 to c4) strong correlation between the appearance/detection of COMP within the cartilage matrix and the stimulation with TNF-α, IL-1β and TNF-α/IL-1β, (d1 to d4) which is dramatically augmented by the co-culture with SFB. (a) Fresh, non-cultured bovine cartilage and (c1) non-stimulated cartilage monocultures do not stain for COMP; in contrast, (b) human OA cartilage shows a positive staining for COMP. (g1 to h4) Immunostaining for the collagen cleavage neo-epitope Col2-3/4C-(short) demonstrates the matrix-degrading capacity of SFB and the amplifying impact of TNF-α, IL-1β and TNF-α/IL-1β on this process. (e) Whereas fresh, non-cultured bovine cartilage lacks signs of collagen cleavage, (f) human OA cartilage exhibits positive staining for the neoepitope. (i to k4) Transmission electron microscopy confirmed the immunohistologically detected collagen breakdown by a decreased optical density of collagen fibres (the dotted line indicates the cartilage surface or the interface between the cartilage and the co-cultured SFB). Magnifications in (a to h4) 200×; inserts 630×; (i to k4) 39,000×.

COMP was barely detectable in fresh, non-cultured cartilage and undetectable in non-stimulated cartilage monocultures. In contrast, faint COMP staining throughout the whole matrix was observed in TNF-α and, in particular, in IL-1β or TNF-α/IL-1β stimulated cartilage monocultures (Figures 4a and c1 to c4).

In contrast, already non-stimulated co-cultures with SFB showed a noticeable staining in the cartilage matrix and SFB (visually even stronger than in cytokine-stimulated monocultures). This staining was further increased by stimulation with TNF-α or, in a more pronounced fashion, with IL-1β and TNF-α/IL-1β (Figures 4d1 to d4). Interestingly, fresh human OA cartilage with its known loss of matrix integrity also exhibited a considerable COMP staining (Figure 4b).

### Detection of collagen cleavage

In fresh, non-cultured cartilage or non-stimulated cartilage monocultures, no staining for cleaved collagen was observed. In contrast, stimulation with TNF-α and IL-1β led to a clear appearance of the collagen cleavage epitope in the extracellular matrix. Collagen cleavage was even more pronounced in TNF-α/IL-1β stimulated cartilage samples (Figures 4e and g1 to g4).

Interestingly, collagen cleavage was already observed in non-stimulated cartilage co-cultured with SFB, indicating the capacity of non-stimulated SFB to degrade cartilage collagen (Figure 4h1). The staining intensity for the collagen cleavage epitope was further increased after stimulation with TNF-α and, in particular, with IL-1β or TNF-α/IL-1β (Figures 4h2 to h4). Fresh human OA cartilage also exhibited a considerable degree of collagen cleavage (Figure 4f).

### Morphological destruction of the cartilage matrix

Transmission electron microscopy showed an organized collagen network with sharp and distinct collagen fibers (rich in contrast) in freshly isolated cartilage or in non-stimulated monocultures (Figure 4i to j1). In contrast, TNF-α, and especially IL-1β or TNF-α/IL-1β stimulated monocultures, showed a clear loss of fibril structure, distinguishable as a decreased contrast of the collagen fibrils (Figure 4j2 to j4).

Even more pronounced destruction was observed in co-cultures with SFB (both non-stimulated and stimulated with TNF-α, IL-1β or TNF-α/IL-1β), in all cases showing a massively reduced optical contrast of the collagen structures in areas near the cartilage surface (Figure 4k1 to k4).

### Invasion of synovial fibroblasts into the cartilage

**Figure 5:**
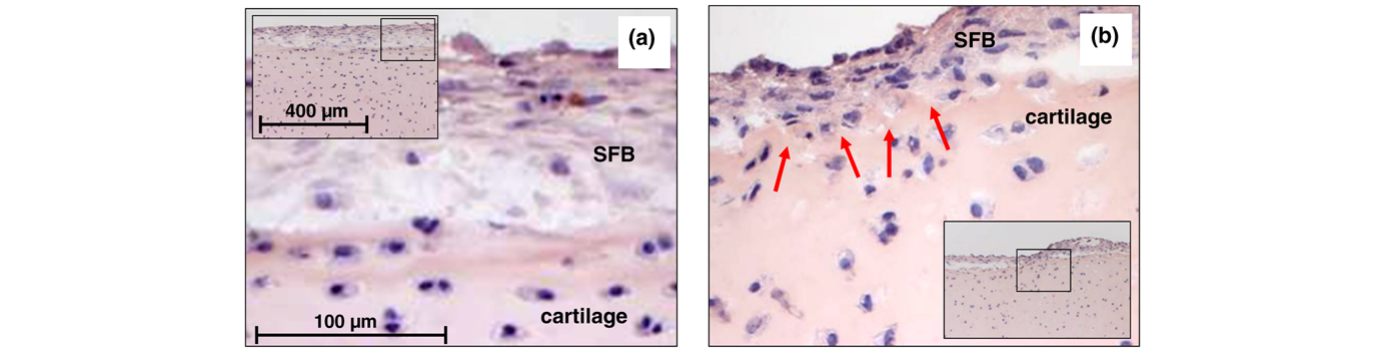
Invasion of cartilage by synovial fibroblasts (SFB) after six weeks of co-culture (Light microscopy). An initial invasion of SFB into superficial cartilage areas was observed in **(b)** TNF-α/IL-1β co-stimulated samples, but not in the case of **(a)** non-stimulated samples and samples stimulated with TNF-α or IL-1β alone (data not shown). Magnifications in (a) and (b) 400×; inserts 100×.

Using light microscopy, an invasive behaviour of co-cultured SFB was not observed in any samples after two weeks (not shown). In contrast, after co-culture for six weeks an initial invasion of SFB into superficial cartilage areas was observed in TNF-α/IL-1β co-stimulated samples (Figure 5b), but not in the case of non-stimulated samples (Figure 5a) and samples stimulated with TNF-α or IL-1β alone (data not shown).

**Figure 6:**
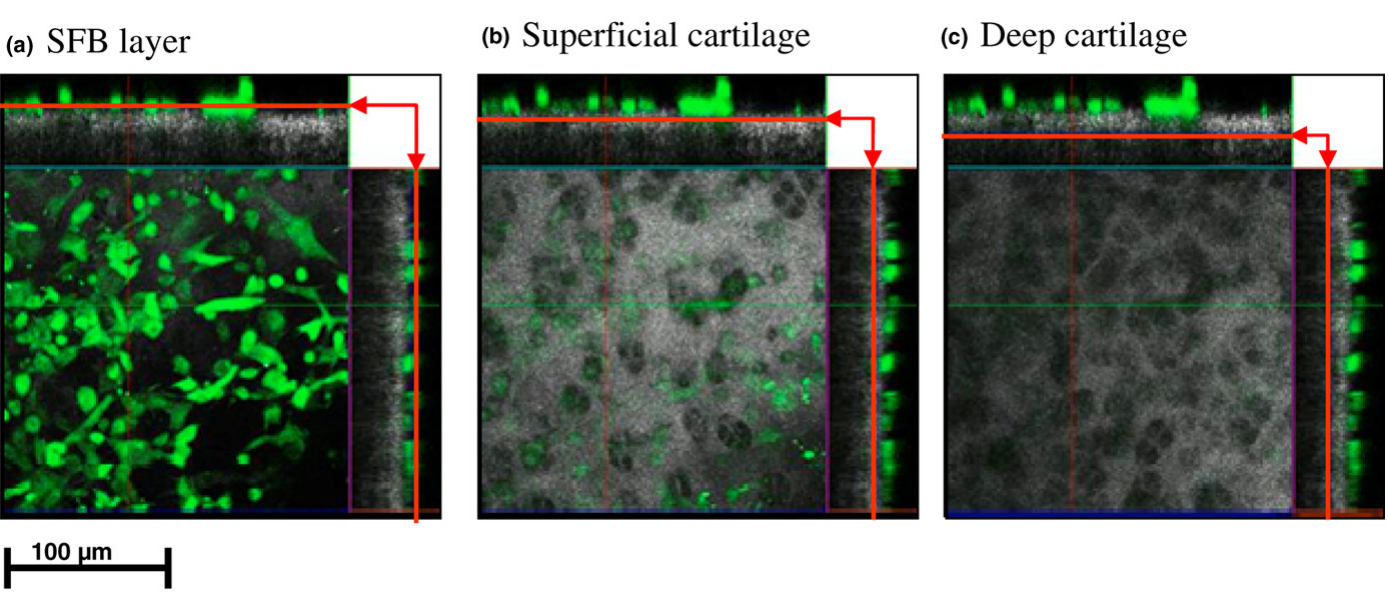
Invasion of cartilage by synovial fibroblasts (SFB) after two weeks of co-culture (Laser scanning microscopy). Erosion of cartilage matrix by fluorescence-labelled SFB was examined in an aqueous setting. Micrographs represent the view from above on the co-culture of cartilage with SFB in different sectional planes (indicated by red arrows) and the corresponding cross-sections. **(a)** plane within the SFB layer on top of the cartilage, **(b)** plane within superficial cartilage and **(c)** plane within deep cartilage zone. Magnifications in 200×.

The attachment of SFB to the cartilage surface and the erosion of cartilage matrix was also analyzed by laser scanning microscopy using SFB fluorescence-labelled before co-culture. Although the SFB layer on top of the cartilage only shows the fluorescence signal of labelled SFB (Figure 6a) and deep cartilage regions only exhibit the reflection signal of the unlabelled cartilage (Figure 6c), the signal in the superficial cartilage consists of both components and therefore indicates an initial invasion of labelled SFB into the cartilage matrix (Figure 6b). This effect was already present in non-stimulated co-cultures and not enhanced by cytokine stimulation (data not shown).

### mRNA synthesis and protein content of COMP in cartilage

**Figure 7:**
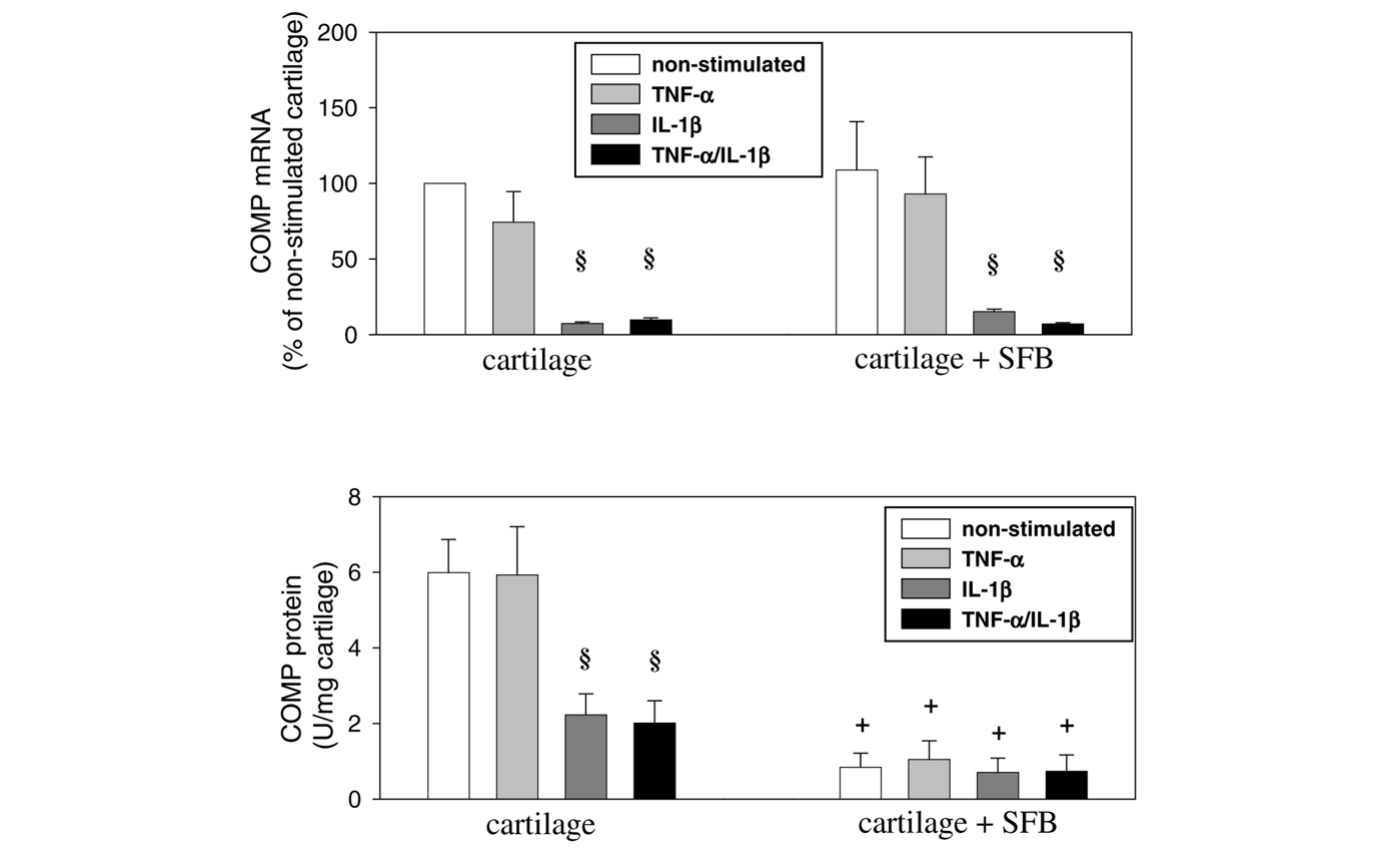
mRNA expression (upper panel) and protein content (lower panel) of bovine cartilage oligomeric matrix protein (COMP). Cartilage from monocultures (n = 5, with two replicates each) and co-cultures with synovial fibroblasts (SFB) (n = 5, with two replicates each) with/without stimulation with TNF-α, IL-1β or TNF-α/IL-1β (14 days) are shown. Gene expression values (means ± standard error of the mean (SEM)), as determined by quantitiative PCR, are expressed as percentage of the values in non-stimulated cartilage monocultures (100%), protein values (means ± SEM) are expressed as units/mg cartilage. § p ≤ 0.05 Mann-Whitney U Test compared with non-stimulated control; + p ≤ 0.05 Mann-Whitney U Test compared with cartilage monoculture.

Stimulation of cartilage monocultures with IL-1β or with TNF-α/IL-1β, but not with TNF-α, significantly reduced the mRNA for COMP compared with the respective non-stimulated control (13- and 10-fold, respectively). Interestingly, the co-culture with SFB had no further influence on the mRNA expression of COMP in cartilage (Figure 7; upper panel).

The reduction of COMP mRNA could be confirmed at the protein level in cartilage monocultures, in which a significant decrease of COMP was detected after IL-1β and TNF-α/IL-1β stimulation (both about three-fold compared with non-stimulated cartilage). Strikingly, a significantly reduced COMP content was observed in all cartilage co-cultures with SFB as compared with the respective monocultures (about three- to nine-fold reduction; Figure 7; lower panel). This further underlines the ability of the co-cultured SFB to fundamentally disturb the cartilage matrix homeostasis. In this case, additional cytokine stimulation seems to play a minor role.

### Analysis of collagen synthesis

**Figure 8:**
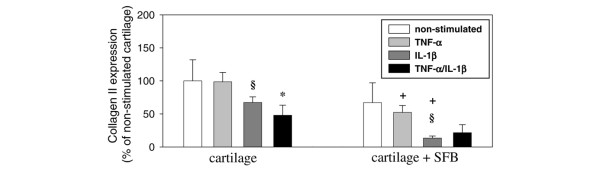
mRNA expression of bovine collagen type II (α1 chain). Cartilage from monocultures (n = 5, with two replicates each) and co-cultures with SFB (n = 5, with two replicates each) with/without stimulation with TNF-α, IL-1β or TNF-α/IL-1β (14 days) were used. Gene expression values (means ± standard error of the mean), as determined by qPCR, are expressed as percent of the values in non-stimulated cartilage monocultures (100%). § p ≤ 0.05 Mann-Whitney U Test compared with non-stimulated control; * p ≤ 0.05 Mann-Whitney U Test compared with stimulation with TNF-α; + p ≤ 0.05 Mann-Whitney U Test compared with cartilage monoculture.

Stimulation of cartilage monocultures with IL-1β (or TNF-α/IL-1β), but not with TNF-α, significantly reduced the mRNA for the α_1 _chain of collagen II (Figure 8). This was also observed in cartilage co-cultures with SFB, in which only IL-1β significantly reduced collagen II expression compared with the non-stimulated co-culture (Figure 8). Notably, both TNF-α and IL-1β stimulated cartilage co-cultures revealed a significantly lower collagen II expression in comparison to the respective monoculture (Figure 8). This indicates that SFB disturb the cartilage homeostasis by both degrading cartilage and suppressing the neosynthesis of collagen II.

### Matrix-metalloproteinase activity

**Figure 9:**
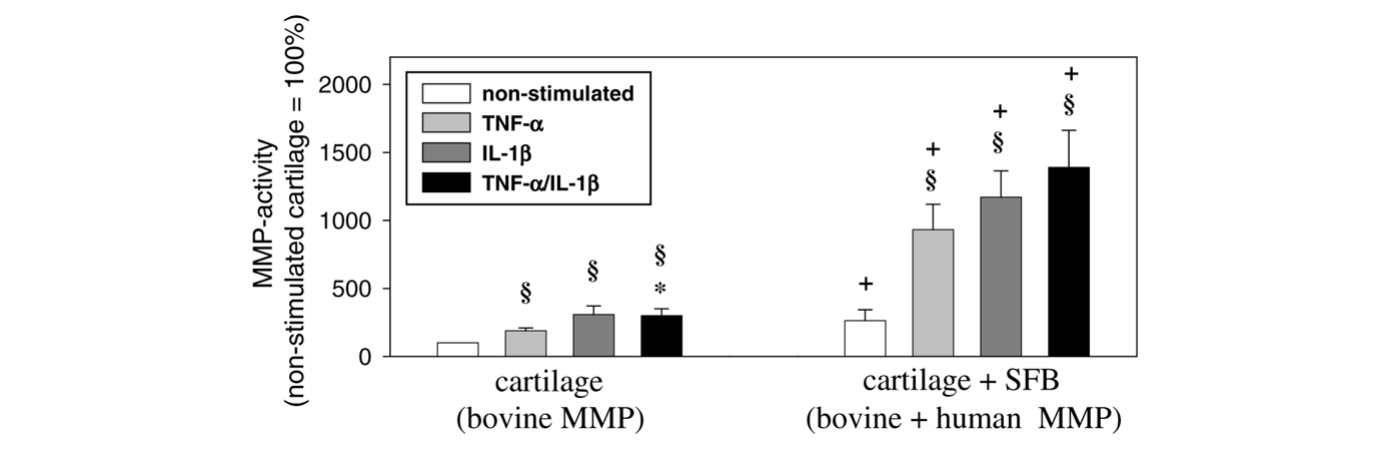
Bovine/human MMP-activity. Supernatants of cartilage monocultures (n = 5, with six replicates each) and co-cultures with synovial fibroblasts (SFB) (n = 5, with six replicates each) with/without stimulation with TNF-α, IL-1β or TNF-α/IL-1β (14 days) were used. Means +/- standard error of the mean are plotted. § p ≤ 0.05 Mann-Whitney U Test compared with non-stimulated control; * p ≤ 0.05 Mann-Whitney U Test compared with stimulation with TNF-α; + p ≤ 0.05 Mann-Whitney U Test compared with cartilage monoculture.

Following activation of latent bovine and human MMP by incubation with APMA, the MMP activity in both cartilage monocultures and co-cultures was significantly increased by stimulation with TNF-α, IL-1β or TNF-α/IL-1β (Figure 9). In addition, all co-cultures with SFB showed a significantly higher MMP activity than the respective monocultures (Figure 9), demonstrating a major contribution of the co-cultured SFB to the secretion of matrix-degrading MMP.

### Caseinolytic activity

**Figure 10:**
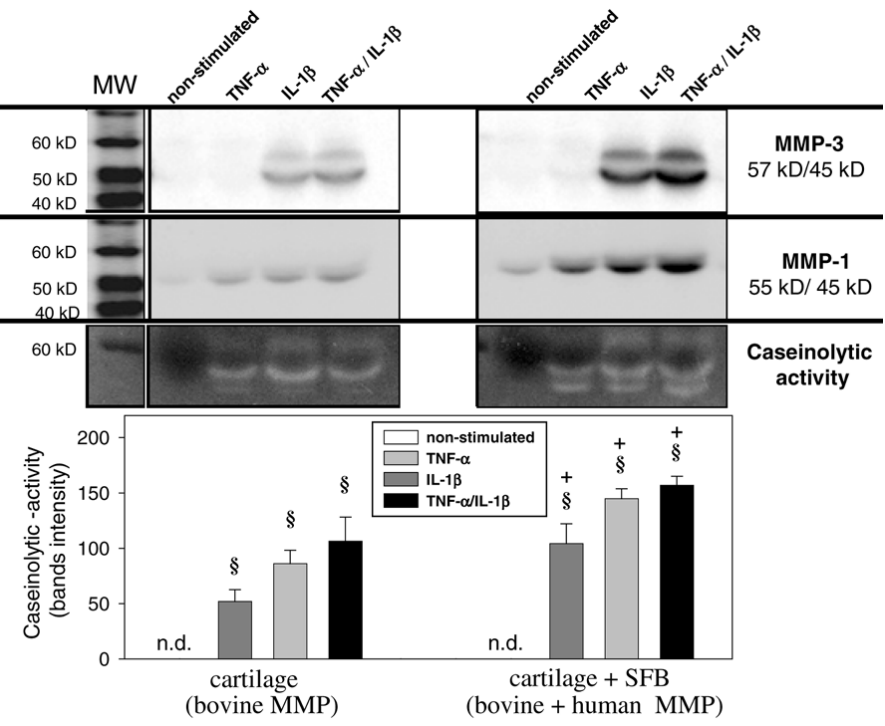
Caseinolytic activity. Supernatants of cartilage monocultures (n = 5, with two replicates each) and co-cultures with synovial fibroblasts (SFB) (n = 5, with two replicates each) with/without stimulation with TNF-α, IL-1β or TNF-α/IL-1β (14 days) were used. In order to assess the total caseinolytic activity (lower panel), the bands for both the active and the latent forms were used for quantification. Means +/- standard error of the mean are plotted. § p ≤ 0.05 Mann-Whitney U Test compared with non-stimulated control; + p ≤ 0.05 Mann-Whitney U Test compared with cartilage monoculture. Parallel analysis of the supernatants by western blot revealed that bovine/human matrix metalloproteases (MMP) 1 and MMP-3 (upper and middle panel) are responsible for the caseinolytic enzyme activity in culture supernatants.

In TNF-α, IL-1β or TNF-α/IL-1β stimulated, but not in non-stimulated, monocultures or co-cultures with SFB, protease bands with caseinolytic activity were detected at a molecular weight of about 45 kD (presumably containing the active forms of MMP-1 and/or MMP-3; Figure 10, lower panel). In TNF-α, IL-1β or TNF-α/IL-1β stimulated co-cultures with SFB, interestingly, additional bands were observed at a molecular weight of about 57 kD, possibly representing the latent form of MMP-1 and/or MMP-3. This was confirmed by immunological detection of both MMP-1 (Figure 10, upper panel) and MMP-3 (Figure 10, middle panel). Successful inhibition of the caseinolytic activity in zymography by EDTA and lack of inhibition by the serine protease inhibitor phenylmethylsulfonyl fluoride further confirmed the MMP character of the bands (data not shown).

In comparison with the respective non-stimulated cultures, the caseinolytic activity in both monocultures and co-cultures was significantly increased by stimulation with TNF-α, IL-1β or TNF-α/IL-1β (Figure 10, lower panel). As in the case of MMP activity, all stimulated co-cultures with SFB showed a significantly higher caseinolytic activity than the respective monocultures (Figure 10, lower panel), further underlining the contribution of the co-cultured SFB.

### Expression of matrix-metalloproteinases

**Figure 11:**
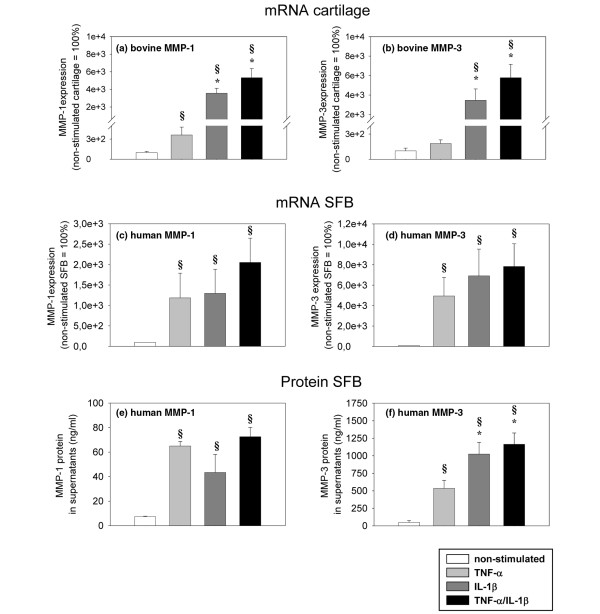
Expression of bovine MMP-1 and MMP-3 mRNA. Cartilage from **(a, b)** monoculture (n = 5, with two replicates each) and **(c, d)** human mRNA/protein in synovial fibroblasts (SFB) after co-culture with cartilage (n = 5, with two replicates each) with/without stimulation with TNF-α, IL-1β or TNF-α/IL-1β (14 days) were used.gene expression values (means ± standard error of the mean (SEM)), as determined by quantitiative PCR, are expressed as percentage of the values in non-stimulated samples (100%). In addition, **(e, f)** the values for human MMP-1 and MMP-3 protein secreted by SFB into the supernatant of co-cultures are shown. The protein levels, as measured in the supernatant by ELISA, are expressed as means +/- SEM. § p ≤ 0.05 Mann-Whitney U Test compared with non-stimulated control; * p ≤ 0.05 Mann-Whitney U Test compared with stimulation with TNF-α.

On the basis of the MMP detected by casein zymography and western blot, the gene expression of MMP-1 and -3 was analysed separately in cartilage derived from monocultures or co-cultures and in SFB obtained from co-cultures. In cartilage monocultures, the level of bovine MMP-1 mRNA was significantly increased by TNF-α stimulation (3.6-fold; Figure 11a), that of bovine MMP-3 mRNA was numerically increased (1.9-fold; Figures 11b). This effect was significantly more pronounced after IL-1β stimulation (36- and 35-fold) or TNF-α/IL-1β stimulation (53- and 58-fold; Figures 11a, b). Notably, there were no significant differences for the gene expression of bovine MMP-1 and MMP-3 between cartilage derived from monocultures or co-cultures (data not shown).

In SFB co-cultured with cartilage, the level of human MMP-1 and MMP-3 mRNA was significantly increased by TNF-α, IL-1β or TNF-α/IL-1β stimulation (12-, 13- and 21-fold, respectively, for MMP-1; 49-, 69- and 78-fold for MMP-3; Figures 11c, d). These results were confirmed at the protein level (by ELISA); the co-cultured SFB secreted significantly more MMP-1 and MMP-3 after stimulation with TNF-α, IL-1β or TNF-α/IL-1β (9-, 6- and 10-fold, respectively, for MMP-1; 11-, 21- and 24-fold for MMP-3; Figures 11e, f).

### Expression of pro-inflammatory cytokines

The influence of the pro-inflammatory cytokines TNF-α and IL-1β on the gene expression of IL-6 and IL-8 was also assessed in cartilage derived from monocultures or co-cultures and in SFB obtained from co-cultures.

**Figure 12:**
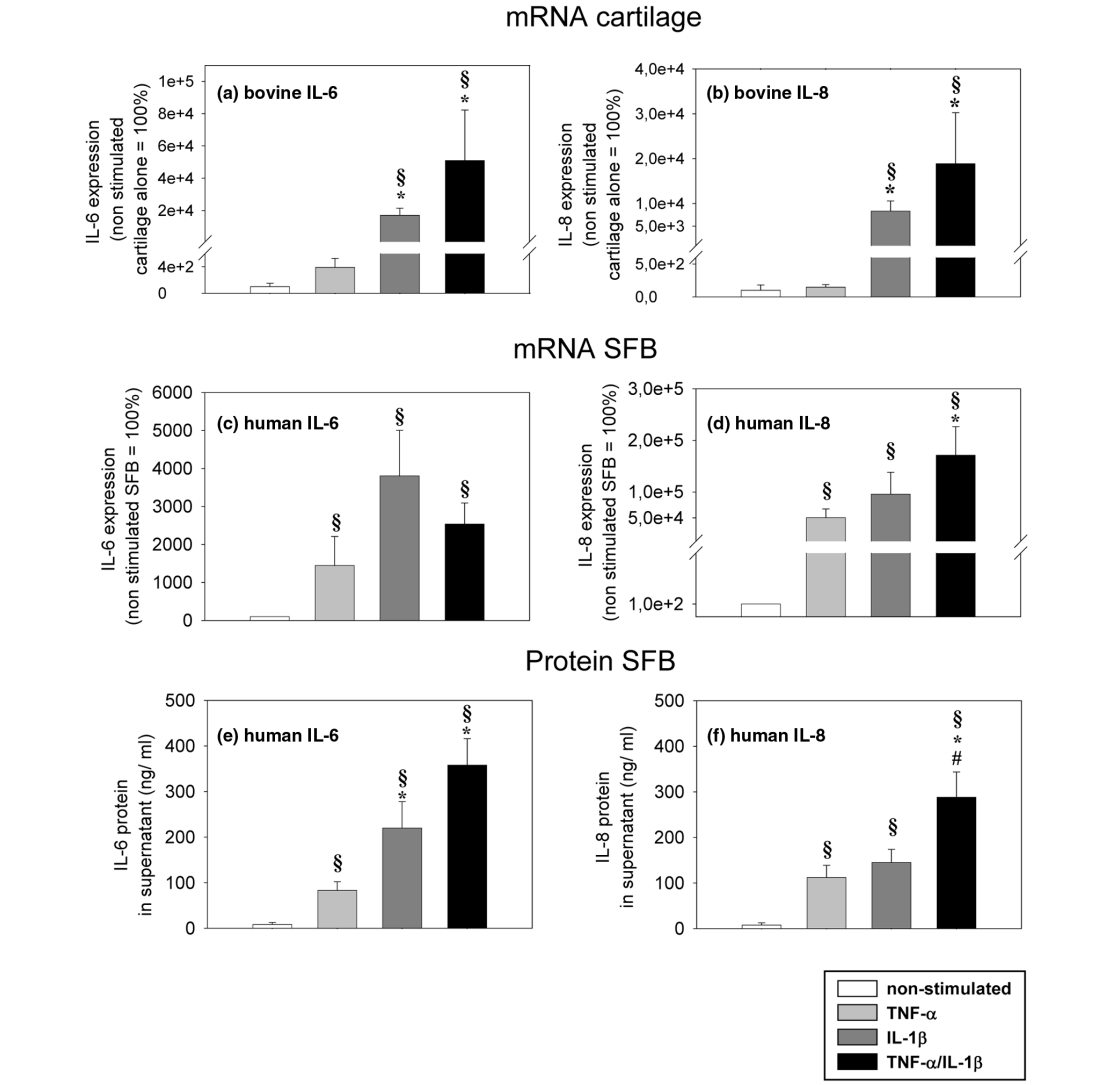
Expression of bovine IL-6 and IL-8 mRNA. Cartilage from **(a, b)** monoculture (n = 5, with two replicates each) and **(c, d)** human mRNA/protein in synovial fibroblasts (SFB) (n = 5, with two replicates each) after co-culture with cartilage with/without stimulation with TNF-α, IL-1β or TNF-α/IL-1β (14 days) were used.gene expression values (means ± standard error of the mean (SEM)), as determined by quantitiative PCR, are expressed as percentage of the values in non-stimulated samples (100%). In addition, **(e, f)** the values for human IL-6 and IL-8 protein secreted by SFB into the supernatant of co-cultures are shown. The protein levels, as measured in the supernatant by ELISA, are expressed as means ± SEM. § p ≤ 0.05 Mann-Whitney U Test compared with non-stimulated control; * p ≤ 0.05 Mann-Whitney U Test compared with stimulation with TNF-α; # p ≤ 0.05 Mann-Whitney U Test compared with stimulation with IL-1β.

In cartilage monocultures, the levels of bovine IL-6 and IL-8 mRNA were exclusively augmented in a significant fashion by IL-1β or TNF-α/IL-1β stimulation (170- and 510-fold for IL-6; 83- and 189-fold for IL-6; Figures 12a, b). As described above for bovine MMP-1 and MMP-3, there were no significant differences for the gene expression of bovine IL-6 and IL-8 between cartilage derived from monocultures or co-cultures (data not shown).

In SFB co-cultured with cartilage, the levels of human IL-6 and IL-8 mRNA were significantly increased by stimulation with TNF-α (14- and 500-fold, respectively), IL-1β (38- and 958-fold, respectively) or TNF-α/IL-1β (25- and 1712-fold, respectively; Figures 12c, d). These results were again confirmed at the protein level; the co-cultured SFB secreted significantly more IL-6 and IL-8 after stimulation with TNF-α (10- and 14-fold, respectively), IL-1β (28- and 19-fold, respectively) or TNF-α/IL-1β (45- and 37-fold, respectively; Figures 12e, f).

## Discussion

### Suitability of the new model

Based on the experimental results described above, our newly-developed *in vitro *destruction model offers several new features in comparison with published *in vitro *models, in that the model uses initially intact cartilage matrix and co-cultured, early-passage SFB with properties close to their *in vivo *features. In contrast, previous models of cartilage destruction mostly worked with either artificial, *in vitro *generated, damaged or devitalised cartilage matrices and were therefore not suitable for examining the process of cartilage destruction in 'healthy' intact cartilage [9-12,20-22]. The mature bovine joints employed in the present study turned out to be a suitable cartilage source, because they are regularly available and are able to harvest up to 80 cartilage discs per joint with standardised, highly homogenous quality. These discs show a completely intact cartilage matrix and surface (no superficial fissures or other OA abnormalities) without primary loss of proteoglycan, both prerequisites for the unequivocal determination of early cartilage alterations. These features are difficult to achieve with human samples because normal human cartilage is usually not available and cartilage from OA or RA patients normally shows drastic signs of matrix destruction/alteration, which often extend from the surface throughout the cartilage matrix. Therefore, models using shaved or cut cartilage surfaces or cartilage with a damaged surface and exposed fibrillar matrix may investigate the progression of pre-existing erosions rather than initial damage [11,13].

The only possible disadvantage of the present system, that is the use of a xenogenic bovine cartilage matrix, may be of minor importance since important matrix molecules (for example collagen II and aggrecan) share a high sequence homology as a result of the close evolutionary relationship between bovine and human; and since human cytokines/chemokines and proteases can also act on bovine chondrocytes or proteins and vice versa (see below; [23]). In addition, limited access to normal human cartilage may allow the validation of selected data in this newly-established *in vitro *cartilage model [24].

Regarding the characteristics of the co-cultured SFB, previous studies have worked with: complete synovial tissue or heterogeneous cell-mixtures (comprised of macrophages, B-cells, T-cells and SFB) [11,13], fibroblasts cell lines or their conditioned supernatants [20,25], and either non-purified, early-passage SFB (possibly still contaminated with macrophages) [9] or late-passage SFB (fourth and higher) [10], known to differ largely from early-passage SFB (first to fourth) [15,26]. The present model, in contrast, uses purified, early-passage SFB with a phenotype similar to their *in vivo *status in the synovial membrane [15], allowing the exact assignment of the observed effects to SFB. Taken together, the present system allows for the first time to simulate the initial cartilage destruction in rheumatoid joints mediated by aggressive SFB. A partial/complete reconstitution of the mixture of inflammatory and mesenchymal cells in the synovial tissue is also possible by adding some or all of the adherent and non-adherent cells obtained during the isolation procedure of SFB [15].

### Destructive processes in the cartilage are induced by co-culture with synovial fibroblasts and are further potentiated by cytokine stimulation

This study shows that non-stimulated RA SFB are capable of rapidly degrading intact undamaged cartilage by inducing a loss of matrix proteoglycan and a cleavage of collagen. The degree of SFB-mediated matrix degradation was further enhanced by stimulation with TNF-α and, in particular, IL-1β or even more pronounced with the combination of TNF-α and IL-1β. Matrix-degrading proteases (MMP and aggrecanases) and pro-inflammatory cytokines (IL-6 and IL-8) in SFB and cartilage were identified as potential direct or indirect mediators for this cartilage destruction. In addition, a suppression of collagen synthesis in chondrocytes by stimulated SFB appears to contribute to the breakdown of cartilage homeostasis.

### Synovial fibroblasts promote cartilage destruction by degradation of extracellular matrix and suppression of matrix synthesis

#### Proteoglycan loss

The limited/absent proteoglycan loss from the cartilage in non-stimulated cartilage monocultures shows that the basic *in vitro *conditions preserve/stabilise the normal cartilage metabolism and render this monoculture a suitable control for cytokine-stimulated monocultures and the respective co-cultures with SFB.

Strong induction of proteoglycan loss in cytokine-stimulated cartilage monocultures points to a major contribution of chondrocytes to cytokine-induced matrix degradation. IL-1β was a stronger inductor of proteoglycan loss than TNF-α, confirming previous results [27-29]. The combination of both cytokines amplified the effect of the respective cytokines alone. This is of particular interest, because the inflamed joint (synovium) is characterised *in vivo *by the concomitant appearance of these pro-inflammatory factors.

Significant enhancement of the proteoglycan loss in non-stimulated or stimulated co-cultures with SFB demonstrates an important role for SFB, whether directly or indirectly via stimulation of chondrocytes. Interestingly, there was a gradient of proteoglycan loss from the cartilage surface to deeper matrix zones, most likely because of the specific zonal properties of cartilage concerning their proteoglycan content [30] or the differential zonal inhibition of matrix/aggrecan neosynthesis by IL-1 (α/β) (data not shown [31]).

#### COMP

COMP was barely or not at all detectable in fresh, non-cultured cartilage or non-stimulated cartilage monocultures, suggesting that COMP, although clearly measurable [32], is masked by other matrix molecules in normal cartilage. Similar results were obtained in fetal human cartilage, which also showed weak pericellular immunoreactivity for COMP [33]. In contrast, COMP staining throughout the whole matrix was observed in TNF-α or IL-1β stimulated monocultures and, in particular, in non-stimulated or stimulated co-cultures with SFB. Increased COMP detection could therefore either reflect a futile, regenerative attempt of chondrocytes [34] or an enhanced demasking/degradation of matrix-bound COMP, although an exclusive connection with the loss of proteoglycans is unlikely because of the incongruent histological results for the two molecules (present study; [34]). Steady-state analysis of the cartilage from monocultures and co-cultures after two weeks showed a substantial reduction of COMP mRNA and protein, at least excluding a successful net reconstitution of COMP. Although the reduction of COMP mRNA was not influenced by co-culture with SFB, the loss of immunoreactive COMP protein from the cartilage matrix was strongly augmented by the SFB, further underlining the contribution of SFB to the disruption of the cartilage matrix homeostasis. Therefore, the COMP appearance in histological sections seems to be a consequence of the initial cartilage destruction and subsequent demasking of this cartilage component. Independent of the precise molecular mechanism, the present study demonstrates for the first time that co-culture of cartilage with RA SFB induces an increased appearance of matrix-bound COMP.

#### Collagen breakdown

Although the above described loss of protective proteoglycans [35] may lead to an enhanced accessibility and cleavage of collagen fibres, the primary collagen cleavage site was not detectable in the supernatant of any experimental group (data not shown). This is most probably based on the fact that cleaved collagen initially remains in the cartilage matrix unless further matrix disaggregation or secondary collagen cleavage occurs [28,36,37]. Indeed, the primary collagen cleavage site was detected immunohistochemically in stimulated (but not non-stimulated) cartilage monocultures, indicating a strong involvement of chondrocytes in the degradation process under pathological conditions. On the other hand, the increased staining intensity in cartilage samples co-cultured with SFB shows that the collagen breakdown was further augmented by SFB, for example via secreted soluble proteinases and/or cytokines.

Electron microscopy confirmed the immunohistochemical results and revealed that severe damage of the collagen network appeared exclusively in stimulated monocultures, as well as in all co-cultures with SFB. Strikingly, all co-cultures showed higher amount of damage than the respective monocultures.

#### Collagen synthesis

In view of an amplification of the potential net loss of collagen from the cartilage matrix, the synthesis of collagen type II (mRNA) was also significantly suppressed in TNF-α and IL-1β stimulated co-cultures. To our knowledge, these are the first data demonstrating a suppressive effect of SFB on collagen type II gene expression in chondrocytes.

### Synovial fibroblasts produce or induce the mediators to destroy cartilage extracellular matrix

#### Aggrecanase activity

The absence of aggrecanase activity (the enzyme predominantly responsible for proteoglycan degradation/depletion [35,38]) in non-stimulated monocultures and co-cultures is consistent with previously reported data [29]. TNF-α and IL-1β are potent inductors of aggrecanase activity in both cases, underlining the key role of these cytokines in proteoglycan depletion. On the other hand, the clearly increased aggrecan activity in co-cultures as compared with monocultures suggests an impact of activated SFB. However, this effect appears to be mediated by the induction of aggrecanase expression in chondrocytes rather than by increased aggrecanase expression in SFB, as indicated by qPCR experiments (data not shown). Alternatively, an activation of matrix-bound aggrecanases by SFB-derived MMP could contribute to the augmented aggrecanase activity in co-culture [39].

#### MMP activity

The present results show a slight, but significant induction of MMP-activity in cartilage monocultures after cytokine treatment, which is further enhanced in co-culture samples with non-stimulated or stimulated SFB. Although the MMP substrate employed in this study can be cleaved by all known MMP, MMP-2 and MMP-9 have the highest rate of turnover for the substrate peptide [17,40], in agreement with their clear detection in the supernatant of all samples by gelatine zymography (data not shown). This may be of pathogenic relevance in RA, because MMP-2 and MMP-9, among other MMP, can further degrade cleaved collagen and thereby support its release from cartilage [41].

#### MMP-1 and MMP-3 expression/activity

Casein zymography, western blots, qPCR and ELISA results indicated that MMP-1 and MMP-3 are detectable at the mRNA, protein and/or activity level (the latter only in stimulated samples) in both cartilage and SFB and the expression of these MMP is further enhanced by either co-culture and/or stimulation with TNF-α, IL-1β or TNF-α/IL-1β. These results are in good agreement with previous reports describing the induction of MMP-1 and MMP-3 in cartilage and SFB by TNF-α and/or IL-1β [42,43]. Therefore, they support the validity of the new model for the analysis of the mechanisms of cartilage destruction by SFB. Both MMP-1, capable of cleaving intact collagen [44], and MMP-3, responsible for the cleavage of other extracellular matrix components [45,46] and the subsequent increase of accessibility of collagen fibrils to other collagenases like MMP-1 [35,42,47], are presumed to be of major importance in the initial joint destruction in the pathogenesis of RA. In addition, MMP-1 is proteolytically activated by MMP-3 [46,48,49], indicating a concerted action of matrix destruction by different MMP.

#### IL-6 and IL-8 expression

Whereas the exposition of SFB to TNF-α and particularly IL-1β or TNF-α/IL-1β led to an enhanced production of the pro-inflammatory cytokines IL-6 and IL-8 (mRNA and protein level), only IL-1β and the combination of IL-1β with TNF-α were capable of inducing IL-6 and IL-8 mRNA in cartilage. As in the case of MMP-1 and MMP-3, these results are in good agreement with previous reports [50,51] and underline the potential importance of these cytokines in the pathogenesis of RA. Concerning IL-6, this is further supported by studies showing that the serum levels of IL-6 correlate with those of C-reactive protein and rheumatoid factors, as well as the degree of joint destruction [52] and that the disruption of IL-6 signalling by receptor-blocking antibodies shows clinical efficacy in RA in phase II clinical trials [2,53,54]. In addition, IL-8 promotes the invasive activity of SFB in co-culture with cartilage slices [22], pointing to a possible connection between IL-8 and cartilage degradation.

#### Invasion of SFB into cartilage

An invasive growth of non-stimulated and stimulated SFB into the superficial cartilage zone was observed after two weeks of co-culture when samples were analysed by laser scanning micoscopy (LSM) (but not by histology), showing that LSM is a suitable and sensitive tool for the analysis of initial stages of cartilage erosion. After co-culture for six weeks the cartilage damage induced by TNF-α/IL-1β stimulated SFB was already detectable by conventional histology, suggesting a somewhat more pronounced superficial cartilage erosion. Thus, SFB (in this case RA SFB) appear capable of invading cartilage within a relatively short time period, in particular if stimulated by pro-inflammatory cytokines such as TNF-α and IL-1β.

## Conclusion

The new *in vitro *model consisting of xenogenic, undamaged bovine cartilage in an interactive culture with human SFB may prove an effective instrument to study the impact of SFB in the initial, early destruction in 'healthy' intact cartilage. This system may be suitable to validate or even partially replace complex animal studies and, in particular, address the importance of isolated, specific synovial cell types in an experimental setting which reflects prominent features of joint destruction in RA. In the long run, the system may allow the testing/screening of the molecular basis and efficacy of new therapeutic strategies and thereby contribute to the improvement of anti-rheumatic therapy.

## Abbreviations

APMA: aminophenylmercuric acetate; CFSE: carboxyfluoroscein succinimidyl ester; COMP: cartilage oligomeric matrix protein; DMEM: Dulbecco's modified eagle medium; ELISA: enzyme-linked immunosorbent assay; FCS: fetal calf serum; H&E: haematoxylin and eosin; HRP: horseradish peroxidase; Ig: immunoglobulin; IL: interleukin; OA: osteoarthritis; PBS: phosphate buffered saline; qPCR: quantitative polymerase chain reaction; RA: rheumatoid arthritis; SDS: sodium dodecyl sulfate; SFB: synovial fibroblast; TNF: tumour necrosis factor.

## Competing interests

The authors declare that they have no competing interests.

## Authors' contributions

D Pretzel established the modified, present form of the model, performed the real-time PCR, the immunohistochemistry and the respective data analyses and wrote the manuscript. D Pohlers performed some of the experiments and participated in writing the manuscript. SW established the initial form of the in vitro destruction model and described it in his diploma thesis. RWK contributed to the design of the study and participated in the layout, writing and finalisation of the manuscript.
